# Semantic Segmentation of Gastric Polyps in Endoscopic Images Based on Convolutional Neural Networks and an Integrated Evaluation Approach

**DOI:** 10.3390/bioengineering10070806

**Published:** 2023-07-05

**Authors:** Tao Yan, Ye Ying Qin, Pak Kin Wong, Hao Ren, Chi Hong Wong, Liang Yao, Ying Hu, Cheok I Chan, Shan Gao, Pui Pun Chan

**Affiliations:** 1 School of Mechanical Engineering, Hubei University of Arts and Science, Xiangyang 441053, China; yantao@hbuas.edu.cn; 2 Department of Electromechanical Engineering, University of Macau, Taipa, Macau 999078, China; mc05403@connect.um.edu.mo (Y.Y.Q.); yc07914@connect.um.edu.mo (L.Y.); 3 Xiangyang Central Hospital, Affiliated Hospital of Hubei University of Arts and Science, Xiangyang 441021, China; angel_angel2345@163.com; 4 Faculty of Medicine, Macau University of Science and Technology, Taipa, Macau 999078, China; hong030427@gmail.com; 5 Shenzhen Institute of Advanced Technology, Chinese Academy of Sciences, Shenzhen 518055, China; ying.hu@siat.ac.cn; 6 School of Medicine, Shanghai Jiao Tong University, Shanghai 200240, China; chancheoki@sjtu.edu.cn; 7 Department of General Surgery, Centro Hospitalar Conde de São Januário, Macau 999078, China; alexchan@ssm.gov.mo

**Keywords:** gastric polyps, semantic segmentation, convolutional neural networks, integrated evaluation approach

## Abstract

Convolutional neural networks (CNNs) have received increased attention in endoscopic images due to their outstanding advantages. Clinically, some gastric polyps are related to gastric cancer, and accurate identification and timely removal are critical. CNN-based semantic segmentation can delineate each polyp region precisely, which is beneficial to endoscopists in the diagnosis and treatment of gastric polyps. At present, just a few studies have used CNN to automatically diagnose gastric polyps, and studies on their semantic segmentation are lacking. Therefore, we contribute pioneering research on gastric polyp segmentation in endoscopic images based on CNN. Seven classical semantic segmentation models, including U-Net, UNet++, DeepLabv3, DeepLabv3+, Pyramid Attention Network (PAN), LinkNet, and Muti-scale Attention Net (MA-Net), with the encoders of ResNet50, MobineNetV2, or EfficientNet-B1, are constructed and compared based on the collected dataset. The integrated evaluation approach to ascertaining the optimal CNN model combining both subjective considerations and objective information is proposed since the selection from several CNN models is difficult in a complex problem with conflicting multiple criteria. UNet++ with the MobineNet v2 encoder obtains the best scores in the proposed integrated evaluation method and is selected to build the automated polyp-segmentation system. This study discovered that the semantic segmentation model has a high clinical value in the diagnosis of gastric polyps, and the integrated evaluation approach can provide an impartial and objective tool for the selection of numerous models. Our study can further advance the development of endoscopic gastrointestinal disease identification techniques, and the proposed evaluation technique has implications for mathematical model-based selection methods for clinical technologies.

## 1. Introduction

Gastric cancer is the fifth most common cancer globally and the third most prominent cause of cancer-related mortality, with more than 782,000 deaths annually [1]. The 5-year survival rate for gastric cancer exceeds 90% if diagnosed at an early stage, but it will decrease to 10–20% if diagnosed at later stages [2]. Gastric polyps show a wide variety of malignant potential and are harbingers of gastric adenocarcinoma. The appearance of gastric polyps may potentially signify a higher risk of intestinal or extra-intestinal malignancy. Depending on histological type, the majority of gastric polyps are categorized as fundic gland, hyperplastic, or adenomatous polyps [3]. In a large pathology study in the United States, the percentage of patients diagnosed with gastric polyps among patients who underwent Esophagogastroduodenoscopy (EGD) and gastric biopsies was 6.35%; 77% were fundic gland polyps, 17% were hyperplastic polyps, and 0.69% were adenomas [4]. Among them, adenomatous polyps have been considered true neoplasms, with malignant transformation occurring in 6% to 47% of cases [5]. Therefore, timely diagnosis, biopsy, and removal of gastric polyps are necessary, which help eliminate the hidden dangers of adenoma early and lower the risk of gastric cancer [6].

Gastric polyps are protuberant lesions originating from the epithelium or submucosa that project into the lumen. Since most gastric polyps are asymptomatic, it is challenging to discover them before a physical examination. EGD has been an effective and direct way to diagnose polyps and perform polypectomy in clinical practice [7]. Endoscopists can examine the situation of the gastric epithelium and treat the polyps through EGD. Nevertheless, the diagnostic accuracy of gastric polyps depends highly on endoscopists and environmental factors. On the one hand, endoscopic characteristics, pathogenesis, histopathological prediction, and management of polyps rely on endoscopists’ experience [7]. Even if experienced endoscopists are involved, operation level and diagnostic rate will be influenced by high workload and fatigue [8]. On the other hand, due to the stomach’s complicated structure and inconsistency in image quality, diagnosis outcomes show large variations in terms of inter- and intra-observer agreement [9].

Computer-aided diagnosis (CAD) systems have been adopted recently to overcome the above challenges [10]. In recent years, CNN-based CAD systems have shown great potential for assisting in the diagnosis of endoscopic polyps [11]. CNN is composed of hierarchical processing, including convolution layers, pooling layers, fully connected layers, et cetera, and can learn the most representation of data with multiple abstractions [12]. Compared with traditional machine learning techniques, CNN can automatically capture spatial and temporal dependencies in images without manual intervention [13]. Image classification, object detection, and semantic segmentation are the essential tasks of using CNN. Differing from image classification and object detection, semantic segmentation can assign each pixel a predefined category label and segment each object region precisely from its background region. Additionally, semantic segmentation can split input images into mutually exclusive subsets and delineate their boundaries accurately, each of which corresponds to a meaningful section of the original image [14]. However, the bounding boxes in object detection are unable to portray the boundaries of polyps precisely. On the contrary, semantic segmentation can accurately delineate the boundaries of polyps and elicit information about their morphology. It is beneficial to clinical diagnosis and treatment because endoscopists must consider polyp topography, size, and endoscopic appearance to decide whether and how to perform polypectomy or whether to enroll in an endoscopic surveillance program when operating EGD [15]. Therefore, an automated CNN-based polyp-segmentation system is developed in this study.

Many semantic segmentation models have been proposed and extensively applied [14]. The selection of the best models for the automated polyp-segmentation system is a complex problem that mainly refers to the performance of the models concerning multiple criteria. Most existing research focuses on improving single-criteria performance without considering integral performance or realistic application. To evaluate the models holistically and select the models with higher performance for the automated polyp-segmentation system appropriately, decision makers (i.e., clinical endoscopists and artificial intelligence researchers) could utilize methodological tools combined with quantitative and qualitative analyses to evaluate the performance of the models. The selection is regarded as a Multiple Criteria Decision Making (MCDM) problem, often arranged as a decision matrix in which alternatives are evaluated with corresponding criteria [16]. After computation, the weights of alternatives that represent the importance of each criterion to each other are obtained and used to calculate the overall weighted score for selection. The subjective method, derived from the decision maker’s judgment, and the objective method, derived by quantifying the intrinsic information of each metric to help eliminate bias and improve objectivity, are the two main MCDM methods [17]. In the selection of clinical assistant techniques, they both are of great importance and should be comprehensively considered to determine the weights and overall score. MCDM methods are capable of dealing with complex situations considering multiple criteria, particularly conflicting criteria, and can facilitate the merger of quantitative and qualitative analyses in a scientific way.

In this study, the diagnosis of gastric polyps is the goal, semantic segmentation is the assisted technology, and MCDM is the evaluation approach, all combining to build an automated polyp-segmentation system to assist endoscopists in the diagnosis of gastric polyps. The main contributions of this study are described as follows:

(1) A high-quality gastric polyp dataset for training, validation, and testing of the semantic segmentation models is built, and the dataset will be publicly available for further research.
(2) This is pioneering research on gastric polyp segmentation. Additionally, seven semantic segmentation models, including U-Net, UNet++, DeepLabv3, DeepLabv3+, PAN, LinkNet, and MA-Net, with the encoders of ResNet50, MobineNetV2, or EfficientNet-B1, are constructed and compared.
(3) The objective and subjective evaluation methods are combined to propose a novel integrated evaluation approach to evaluate the experimental results, aiming at the determination of the best CNN model for the automated polyp-segmentation system.

The remaining sections are organized as follows: Section 2 reviews several existing studies about CNN-based gastric polyp diagnosis and some MCDM methods. Section 3 introduces the collected gastric polyp dataset, the semantic segmentation models used in this paper, and the novel integrated evaluation approach. Section 4 introduces the implementation details and test results of segmentation models with different encoders. An in-depth discussion of the result is also presented. Section 5 concludes and provides our future research plan.

## 2. Related Work

### 2.1. CNN-Based Gastric Polyp Diagnosis

In this section, a brief review of CNN-based gastric polyp diagnosis is summarized. Zhang et al. [18] developed a CNN for gastric polyp detection based on the Sigle Shot MultiBox Detector (SSD). It can achieve real-time detection speed with 50 frames per second (FPS), and the mean average precision (mAP) is increased by 90.4%. Not only did they pioneer research on gastric polyp detection, but they also published their dataset of 404 endoscopic images with gastric images. Laddha et al. [19] applied YOLOv3 and YOLOv3-tiny for gastric polyp detection. They obtained a mAP of 0.91 and a mAP of 0.82. Wang et al. [20] used an improved Faster R-CNN network to detect gastric polyps and obtained a precision of 78.96%, a recall rate of 76.07%, and an F1-score of 77.49%. Cao et al. [21] developed the YOLOv3 network, which combined a feature extraction module and a self-developed fusion module, to help with small gastric polyp detection. The improved network obtained a decent performance with an F1 score of 88.8%. Durak et al. [22] collected 2195 endoscopic images consisting of 3031 polyp labels retrospectively and implemented them on YOLOv4, CenterNet, EfficientNet, and the other five existing models. The YOLOv4 model showed the best performance, with a mAP of 87.95%.

As summarized in Table 1, up to now, the existing papers on the diagnosis of gastric polyps are all based on CNN, but only object detection is performed; the function of semantic segmentation has not been available yet. Moreover, the evaluation metrics are solitary without adequate consideration of clinical requirements. In addition to accuracy, metrics of other aspects (e.g., speed and number of model parameters) should also be considered to evaluate the performance of the model.

Likewise, Zhang et al. [23] introduce a lightweight transformer detection head and a plain FPN into the original YOLOX, which obtain a precision of 62% for erosion and a precision of 77% for ulcers in their own dataset that contains 2074 images. But their model obtains a precision of 99.72% in colon polyp detection in the public dataset. YOLOX is robust in multiple-object-tracking tasks and has a very competitive inference speed. YOLOX is a new generation of high performance and speed in the field of object detection, which shows great potential in gastric polyp detection and other lesion detection in endoscopy.

### 2.2. MCDM Methods

Numerous MDCM methods have been proposed in the literature and widely applied in different fields since 1970 [24]. MCDM methods are usually designed for specific cases and have their pros and cons. Some typical MCDM methods are reviewed for deeper comprehension. The Analytic Hierarchy Process (AHP) is well-known and widely used [25]. It is a structured technique for organizing and analyzing complex decisions based on mathematics and psychology. Another technique called the analytic network process method is available. This method is generalized from AHP and allows modeling the interaction and dependencies between criteria [26]. In [27], a decision-making trial and evaluation laboratory approach was discussed, which analyzes the interdependent relationships among factors in a complex system and ranks them for long-term strategic decisions made by considering experts’ judgments. In addition, a method called Technique for Order Preference by Similarity (TOPSIS) is also available [28]. TOPSIS uses the Euclidean distance to determine the relative proximity of an alternative to the optimal solution. An alternative priority order can be determined by comparing relative proximity. In the above MDCM methods, every method shows a unique specialty and different advantages. This article utilizes one of them to evaluate the CNN models.

## 3. Materials and Methods

### 3.1. Dataset

Public datasets for gastric polyps are lacking. The sole public dataset offered by Zhang et al. [18] only contains 354 gastric polyp images. The number is insufficient, so we create our gastric polyp dataset to train and test the semantic segmentation models. This single-center research was approved by the ethics committees of the Xiangyang Center Hospital (XCH) and followed the Declaration of Helsinki [29]. As a retrospective study, written informed consent was waived. The patient’s information (e.g., name, I.D. number, etc.) was removed from the original EGD images to maintain confidentiality. The endoscopists searched the cases containing the diagnosis of gastric polyps in the medical record database of the endoscopy center from January 2017 to January 2021. From 1428 patients, 10,596 endoscopic images were initially collected and screened by a junior endoscopist. The original images underwent further screening to obtain high-quality images. The exclusion criteria are as follows:

(1) Images that used endoscopic optics other than standard white-light endoscopy.
(2) The anatomical position of the image is not in the stomach (like the esophagus).
(3) Images that contain no polyps.
(4) Images that are damaged and low-quality due to halation, mucous, blurring, lack of focus, low insufflation of air, et cetera.

**Figure 1:**
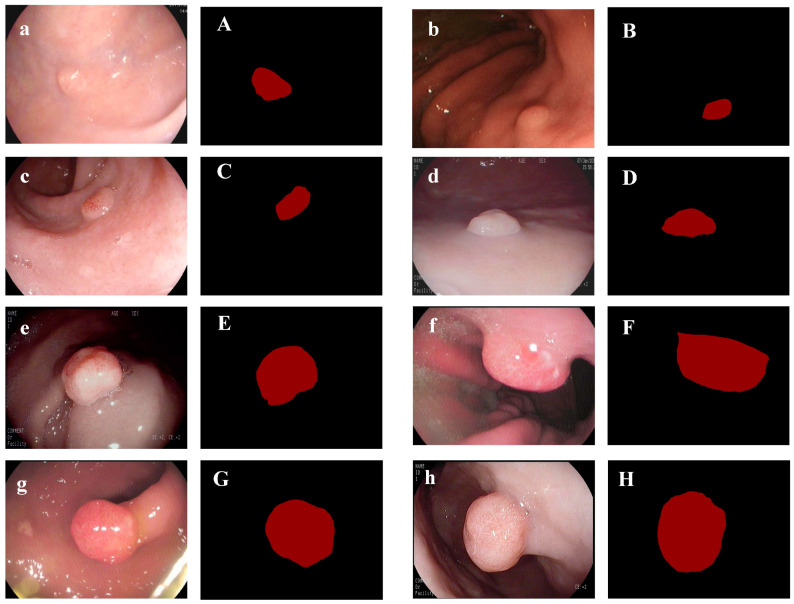
Original images of gastric polyps (i.e., (**a**–**h**)) and corresponding ground-truth masks (i.e., (**A**–**H**)) in the dataset.

After two rounds of selection and rechecking, 487 images from 148 patients were acquired. Their sizes vary from 1296 × 1084 pixels to 356 × 302 pixels since they were taken on different devices. To ensure the labels of polyps were correct, annotation was performed by two endoscopists using Lebelme software. Endoscopists outline the edges of the gastric polyps in the endoscopic images. After processing, the corresponding ground-truth masks are generated, which are the visual representation of manual segmentation. In the masks, all pixels are classified into two categories. As shown in Figure 1, the labels of all pixels in the red part of the masks are the gastric polyps, and the labels of the pixels in the remaining part are the background. The dataset is divided into three sections with a ratio of 8:1:1, which are used for training, validation, and testing.

It is noteworthy that the endoscopic images in the dataset all contain polyps. Normal stomach endoscopic frames are the main part of most live endoscopic videos, which are excluded, leading to a sharp plummet in the number of images in the dataset. The reason why normal stomach endoscopic images are excluded is due to the performance of the semantic segmentation models and clinical consideration. For model performance, worse generalization will occur if the training images contain normal stomach endoscopic images [30]. For clinical considerations, a higher false positive (FP) rate is more desirable than a high false negative (FN) rate since the system’s sensitivity to polyps can help the endoscopists focus more on the suspicious lesions. In terms of the CAD system, the benefit of a misdiagnosis outweighs the benefit of a missed diagnosis [30].

### 3.2. Existing Semantic Segmentation Methods

**Figure 2:**
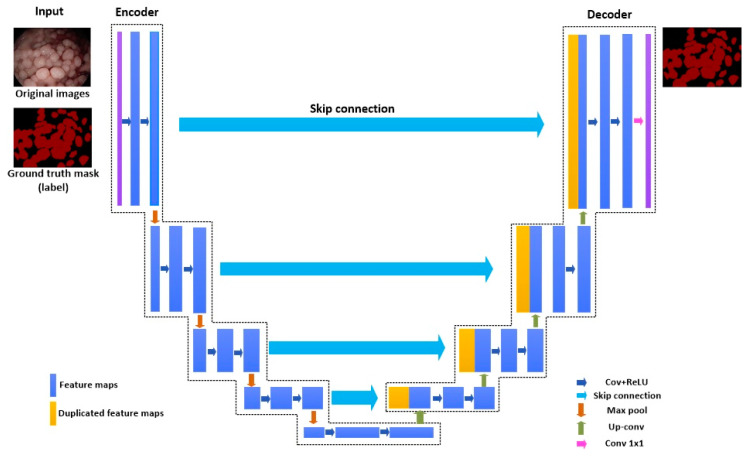
Architecture of U-Net.

Various semantic segmentation algorithms have proven excellent results using CNN in natural images [14]. Generally, CNN can be trained to learn a mapping from original images to ground-truth masks through successive operations, such as convolution, pooling, and upsampling. In this paper, some classical semantic segmentation models, including U-Net, UNet++, DeepLabv3, DeepLabv3+, PAN, LinkNet, and MA-Net, are applied with different encoders to accomplish end-to-end semantic segmentation of gastric polyps [31,32,33,34,35,36,37]. To avoid a dull description, a general U-Net is utilized to explain the idea of most semantic segmentation models, and its architecture is shown in Figure 2. The encoder will usually decrease the spatial resolution and extract the image features, in which convolution and max-pooling are essential to achieve feature extraction, while the symmetric decoder will upsample to the original input resolution and result in low-dimension predictions. In the symmetric decoder, the feature maps are concatenated from the upsampling and the skip connection. The skip connections in U-Net are valid and innovative, increasing and compensating for the semantic information from upsampling.

In other models, the essence of U-Net remains, but with varying constructions, convolution operations, upsampling methods, or combinations with others, such as deep supervision and attention mechanisms. Among the various models adopted in this study, as summarized in Table 2, each has its own strengths and weaknesses. U-Net takes a simple structure and consumes a small amount of training data, but an unchanged structure makes it difficult to find the optimal structure. UNet++ takes advantage of redesigned skip pathways and deep supervision, which improve the segmentation quality of varying-size objects, but it is time-consuming and easily prone to error. Both Deeplabv3 and Deeplabv3+ suffer from the gridding effect problem, though Deeplabv3+ can capture smaller objects and has a more defined boundary. PAN is light and has a low computational cost, but it is difficult to locate small objects precisely. LinkNet is real-time-oriented but requires a large amount of training data and produces inaccurate results. Although MA-Net can obtain good performance in many cases, it is too complex and time-consuming [31,32,33,34,35,36,37]. Given the different strengths and weaknesses of each model, extensive experiments are necessarily required to select the optimal model.

### 3.3. Integrated Evaluation Approach

Each model has its own potential and drawbacks. It is arduous to select the most optimal model for application in the context of multiple criteria, especially when some are mutually exclusive. Thus, it is necessary to propose a specific evaluation approach to evaluate the performance of semantic segmentation comprehensively.

**Figure 3:**
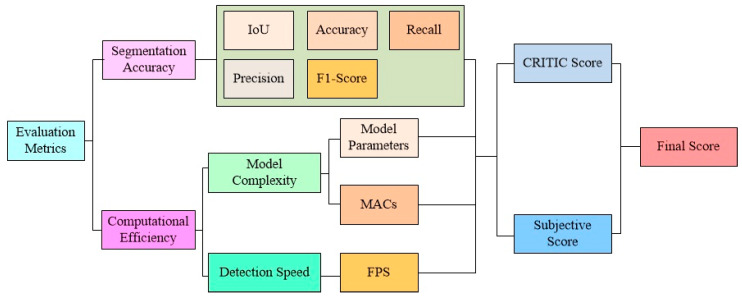
Systematic design of evaluation metrics.

Complex systematic metrics based on the MCDM method are newly proposed. Figure 3 shows the dendrogram of systematic metrics. The dimensions of first-level metrics are segmentation accuracy and computational efficiency. For segmentation accuracy, Intersection over Union (IoU), accuracy, recall (i.e., sensitivity), precision, and F1-score were utilized as the specific metrics following the recommendation from [38]. The thresholds are all set to 0.5. The experiments belong to pixel–binary classification tasks. According to the realistic category and prediction results, pixel results are divided into true positive (TP), FP, true negative (TN), and FN. IoU calculates the degree of similarity between the predicted masks and the ground-truth masks. IoU varies from 0 to 1 (0–100%), with 0 or 0% indicating no overlap (i.e., the worst) and 1 or 100% indicating perfect segmentation. Accuracy is the percentage of pixels categorized correctly in the endoscopic image. Recall measures the proportion of the pixels labeled as polyps correctly recognized. Precision measures the proportion of the pixels correctly labeled as polyps. The F1-score is the harmonic mean of accuracy and recall. Table 3 shows the calculation equations for the above metrics.

Computational efficiency is a property of an algorithm that refers to the number of computational resources and running time used by the algorithm, divided into the third-level metrics of model complexity and detection speed [39]. To measure the complexity of a model, the number of model parameters and multiply–accumulate operations (MACs) are used. The model parameters in Figure 3 are the sum of the numbers of weights and biases on CNN, which stands for the number of parameters that will be learned during forward inference and backward learning. MACs compute the product of two numbers and add that product to an accumulator physically [40]. A MAC is one multiplication and one addition, which can each be a floating point operation (FLOP), i.e., 1 MAC counts as roughly 2 FLOPs. Most modern hardware architectures use the fused multiply–add instruction for operations with tensors, and MACs follow the instruction directly [40]. As a result, MACs are preferred over FLOPs in this paper. To evaluate detection speed, frames per second (FPS) are calculated by using the mean inference time per image in the test dataset [41].

In MCDM, the rank is determined by the overall weighted score, which will help determine the most optimal model. Hence, an integrated evaluation approach is presented that takes the quantitative and qualitative analyses in the context of MCDM into account. The integrated evaluation approach combines an objective method with a subjective method. Diakoulaki et al. [42] proposed the method entitled Criteria Importance Through Intercriteria Correlation (CRITIC) for the verification of objective weights of comparative importance in financial analysis. In comparison to other objective methods, the CRITIC method, by incorporating both contrast and intensity conflict, provides perception into the quiddity of the quandary generated in the structure of the MCDM problem. It gives the ideal values of cost and benefit criteria concurrently to normalize the decision matrix, but the other methods handle them individually. Moreover, it is the first method that calculates the similarity of the criteria with the correlation coefficient among the criteria. After applying the CRITIC method to the metrics, the objective weights and corresponding objective scores of the models are obtained. The CRITIC method consists of the following steps:

There are n models to be evaluated and p metrics to form the original index matrix:

(1) $$X=\left(\begin{array}{ccc} x_{11} & \ldots & x_{1p}\\ \vdots & \ddots & \vdots \\ x_{n1} & \cdots & x_{np} \end{array}\right)$$

where $x_{ij}$ means the value of the jth metric of the ith model. Dimensionless processing should be used for all metrics to reduce the influence of different dimensions on evaluation outcomes.

(2) $$\left\{\begin{array}{c} benefitmetric:x_{ij}^{\prime }=\frac{x_{j}-x_{min}}{x_{max}-x_{min}}\\ costmetric:x_{ij}^{\prime }==\frac{x_{max}-x_{j}}{x_{max}-x_{min}} \end{array}\right.$$

The contrast intensity of the corresponding criterion can be expressed as standard deviation $\sigma_{j}$. Conflict between factors can be expressed as a correlation coefficient, which is calculated using Equation (3).

(3) $$R_{j}=\sum_{i=1}^{P}\left(1-r_{ij}\right)$$

where $r_{ij}$ is the Spearman rank correlation coefficient. The amount of information $C_{j}$ emitted by the jth metric can be determined by composing the measures that quantify the two notions using Equation (4).

(4) $$C_{j}=\sigma_{j}\sum_{i=1}^{P}\left(1-r_{i^{j}}\right)=\sigma_{j}R_{j}$$

Objective weight is calculated by normalizing these values to unity using Equation (5).

(5) $$\omega_{j}=\frac{C_{j}}{\sum_{j=1}^{n}C_{j}}$$

So, the CRITIC score is obtained using Equation (6).

(6) $$S_{i}=\sum_{j=1}^{n}\omega_{j}x_{ij}^{\prime }$$

The objective method is not totally perfect because the expert’s experience always plays a crucial role in clinical treatment. Experienced experts tend to be instinctive in problem-solving and more efficient at dealing with complex emergencies, and patients intend to trust experienced experts more as a result. The subjective weighting of experts should be used in the selection of models. The weights of the subject method are derived from experienced endoscopists from XCH and some medical doctors in Mainland China and Macau, as shown in Table 4. The coefficient of each metric means the relative importance of all metrics, which depends mostly on clinical needs. IoU and FPS occupy the largest proportion because making a clinical diagnosis accurately and quickly is a fundamental requirement for endoscopists, as is the automated polyp-segmentation system.

Therfore, the scores of the subjective method are calculated as follows:

(7) $$S_{i,sub}=x_{i,IoU}^{\prime }\times 0.3+x_{i,ACC}^{\prime }\times 0.05+x_{i,RE}^{\prime }\times 0.05+x_{i,PR}^{\prime }\times 0.05+x_{i,F1}^{\prime }\times 0.05+x_{i,Params}^{\prime }\times 0.1+x_{i,MAC}^{\prime }\times 0.1+x_{i,FPS}^{\prime }\times 0.3$$

where $S_{i,sub}$ denotes the subjective score of the models. $x_{i,IoU}^{\prime }$, $x_{i,ACC}^{\prime }$, $x_{i,RE}^{\prime }$,$x_{i,PR}^{\prime }$, $x_{i,F1}^{\prime }$, $x_{i,Params}^{\prime }$, $x_{i,MAC}^{\prime }$ and $x_{i,FPS}^{\prime }$denotes normalized results of IoU, accuracy, recall, precision, F1-score, number of parameters, number of MACs, and FPS, respectively.

The final quantitative score is obtained by combining the subjective and objective evaluation methods. It is calculated as follows:

(8) $$S_{final}={\delta S}_{i}+{\mu S}_{i,sub};\delta +\mu =1$$

where $S_{i}$ stands for the CRITIC score. $S_{final}$denotes the final score. The coefficients δ and μ are used to determine how much importance should be assigned to objective and subjective methods. δ=μ=0.5 is employed in our research for comprehensive consideration. Therefore, the best semantic segmentation model can be settled by rank, and the “best score” model is finally selected as the core algorithm of the proposed automated polyp-segmentation system.

## 4. Experiments and Results

### 4.1. Experimental Configuration

Transfer learning is flexible and robust; a pre-trained model can be extracted as the weights for a model on a new task. All semantic segmentation models were pre-trained on ImageNet to improve their prior knowledge [43]. The hyperparameters were defined empirically. The Adam optimization algorithm was employed for the optimization of the network with a learning rate of 0.0008 and a batch of 4 images with 80 epochs [44]. Random rotation, random horizontal flipping, and random vertical flipping were utilized for data augmentation, while the input resolution was resized to 256 × 256 using the bicubic interpolation method [45]. The loss function is of paramount importance because it triggers the backward learning process in model training. Some loss functions are designed to alleviate the problem of data imbalance, of which the Dice loss function is a typical one. Dice loss is a kind of region-based loss, which means that the loss value and gradient of a pixel are not only related to the label and predicted value of this pixel but also related to the labels and predicted values of other pixels. As the Dice loss function tends to dig for polyp areas, it is selected as the loss function in the training process [46]. ResNet50, MobileNetV2, and Efficient-B1 were used as the encoders due to their powerful feature extraction ability and inference speed [47,48,49]. Table 5 lists the specifications of the workstation and environmental configurations. To ensure fair-minded experiments, all of the models were trained on the same workstation with the same hyperparameters.

### 4.2. Results

Feature extraction networks are decisive for pixel classification in semantic segmentation [50]. Therefore, three different advanced CNNs (i.e., ResNet50, MobileNetV2, and EfficieNet-B1) were used as the encoders. Among the ResNet series algorithms, ResNet50 is a fast and accurate approach that is frequently employed as the backbone of deep neural networks. Compared with the deeper networks (e.g., ResNet101 and ResNet152), ResNet50 has a speed advantage. Compared with the shallower networks (e.g., ResNet18 and ResNet34), which consume less storage and computational power, ResNet50 can achieve better training results due to its better feature extraction ability. Therefore, the ResNet50 algorithm was chosen as the encoder based on the requirements of the polyp segmentation task, taking into account both speed and precision [51]. Table 6 shows the quantitative results and evaluation scores for the test dataset in the experiments.

In the class of U-Net in Table 6, the U-Net with the encoder of EfficientNet-B1 obtains the best performance in segmentation accuracy, with an IoU of 96.53%, an accuracy of 98.14%, a recall of 98.16%, a precision of 98.12%, and an F1-score of 98.14%, respectively. However, its detection speed is 22 FPS, which is the slowest in the U-Net experiment. When using ResNet50 as the encoder, the number of model parameters and the number of MACs of U-Net are several times that of the others, which requires more storage and computational power. Moreover, its segmentation accuracy is the worst in this class. The number of model parameters in UNet++ is greater than that of U-Net, but UNet++ models can almost improve segmentation accuracy as compared with U-Net. In the class of UNet++, UNet++ with the encoder of EfficientNet-B1 obtains the best segmentation accuracy, but its detection speed is 21 FPS, which is the slowest. Although UNet++ with the encoder of MobileNetV2 achieves unremarkable performance, it creates a trade-off between segmentation accuracy and computational efficiency. It obtains the highest score in both the CRITIC method and the subjective method. Moreover, its final score not only far outperforms other U-Net++ models but also achieves the best score of all semantic segmentation models in the overall experiment.

DeepLabv3 encoded with ResNet50 obtains the best performance in segmentation accuracy in the class of DeepLabv3. The IoU, accuracy, recall, precision, and F1-score are 96.23%, 97.95%, 97.98%, 97.93%, and 97.96%, respectively. However, it has a large model complexity. DeepLabv3 with the encoder of MobileNetV2 obtains the highest final score of all DeepLabv3 models, which implies that the overall performance is more satisfactory. DeepLabv3+ with the encoder of MobileNetV2 obtains an outstanding result in terms of detection speed, with 27 FPS. However, the IoU, accuracy, recall, precision, and F1-score are 95.22%, 97.36%, 97.42%, 97.32%, and 97.37%, respectively. DeepLabv3+ with the encoder of ResNet50 obtains a competitive score in both the CRITIC method and the subjective method, but its model complexity is a big concern.

In the overall result of Table 6, PAN obtains the best performance in model complexity when using Mobilenet-v2 as the encoder. However, it obtains poor results in segmentation accuracy, with an IoU of 91.83%, an accuracy of 95.09%, a recall of 98.86%, a precision of 92.71%, and an F1-score of 95.52%, respectively. PAN with the encoder of ResNet50 has obvious advantages in segmentation accuracy and detection speed as compared with the other two semantic segmentation models in this class. Although it has a large model complexity, the final score is still the highest out of the three models.

When using EfficientNet-B1 as the encoder, LinkNet obtains the best performance in segmentation accuracy, with an IoU of 96.32%, an accuracy of 98.04%, a recall of 98.08%, a precision of 98.00%, and an F1-score of 98.04%. Although it has a small model complexity, its detection speed is slower than the other two models in this class. LinkNet with the encoder of ResNet50 obtains the highest final score in this class because of its outstanding segmentation accuracy and detection speed.

In the class of MA-Net in Table 6, MA-Net obtains the best performance in segmentation accuracy and model complexity when using EfficineNet-b1 as the encoder. Specifically, the IoU, accuracy, recall, precision, F1-score, number of model parameters, and GMACs are 96.57%, 98.15%, 98.16%, 98.14%, 98.15%, 11.6 M, and 2.41, respectively. However, its detection speed is too slow, resulting in an unremarkable final score for the model. MA-Net with the encoder of ResNet50 and MA-Net with the encoder of EfficineNet-b1 obtain outstanding results in segmentation accuracy, but they show obvious flaws in detection speed.

The semantic segmentation models show their own pros and cons from a qualitative perspective when compared with each other. Due to the simplicity of U-Net’s topology, its GMACs are small but nevertheless produce a decent result in segmentation accuracy. UNet++ strikes a balance between segmentation accuracy and computation efficiency and obtains excellent results in both metrics. DeepLabv3 and DeepLabv3+ yield results that are nearly identical, although DeepLabv3+ has a slightly faster detection speed. Although PAN and LinNet are both lightweight and necessitate fewer computational resources, it seems difficult to locate the polyps precisely, yielding subpar segmentation accuracy. MA-Net is very heavy and expensive in computational resources, although it obtains the best result in segmentation accuracy.

**Figure 4:**
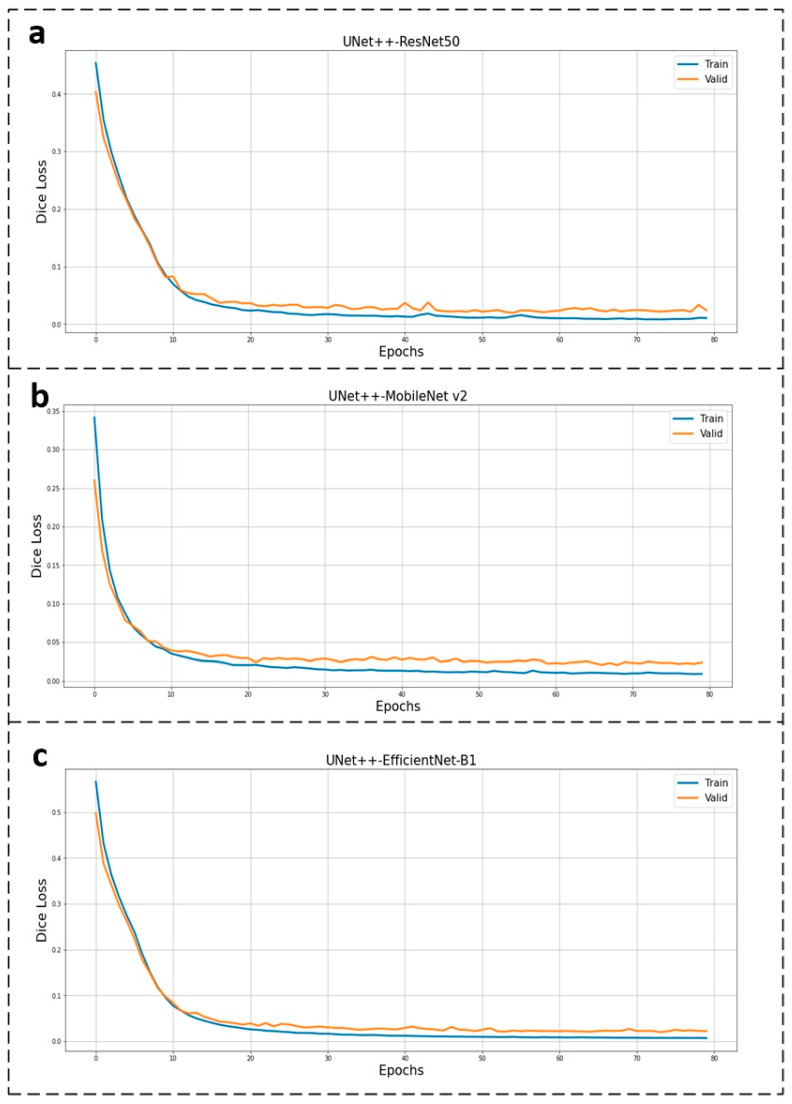
Dice loss curve of U-Net++ with different encoders. (**a**) Dice loss curve of U-Net++ with the encoder of ResNet50; (**b**) Dice loss curve of U-Net++ with the encoder of MobineNet v2; (**c**) Dice loss curve of U-Net++ with the encoder of EfficientNet-B1.

Each model has its own characteristics based on its quantitative metrics. In general, models with high segmentation accuracy are usually complicated to achieve high computational efficiency. Correspondingly, models with high computational efficiency usually tend to have low segmentation accuracy. Model comparison is extremely challenging when there are conflicts between the metrics. The proposed integrated evaluation approach provides a measurable score for comparison. Table 6 shows that UNet++ with the encoder of MobineNet v2 obtains the highest points in the CRITIC score, subjective score, and final score. It means that no matter the aspect of quality, quantity, or a combination of both, the comprehensive performance of the model is the best. In other words, a trade-off between segmentation accuracy and computational efficiency has been achieved. Moreover, the detection speed of 26 FPS shows that the goal of real-time segmentation has been achieved. Figure 4 shows that the Dice loss curve converges quickly and smoothly, which implies that the training process can converge to the global minima with no sign of overfitting or underfitting. The complexity of this model is appropriate for the dataset and is neither overly complex nor overly simplistic. After all considerations, UNet++ with the encoder of MobineNet v2 is chosen as the final model for the automated poly-segmentation system.

**Figure 5:**
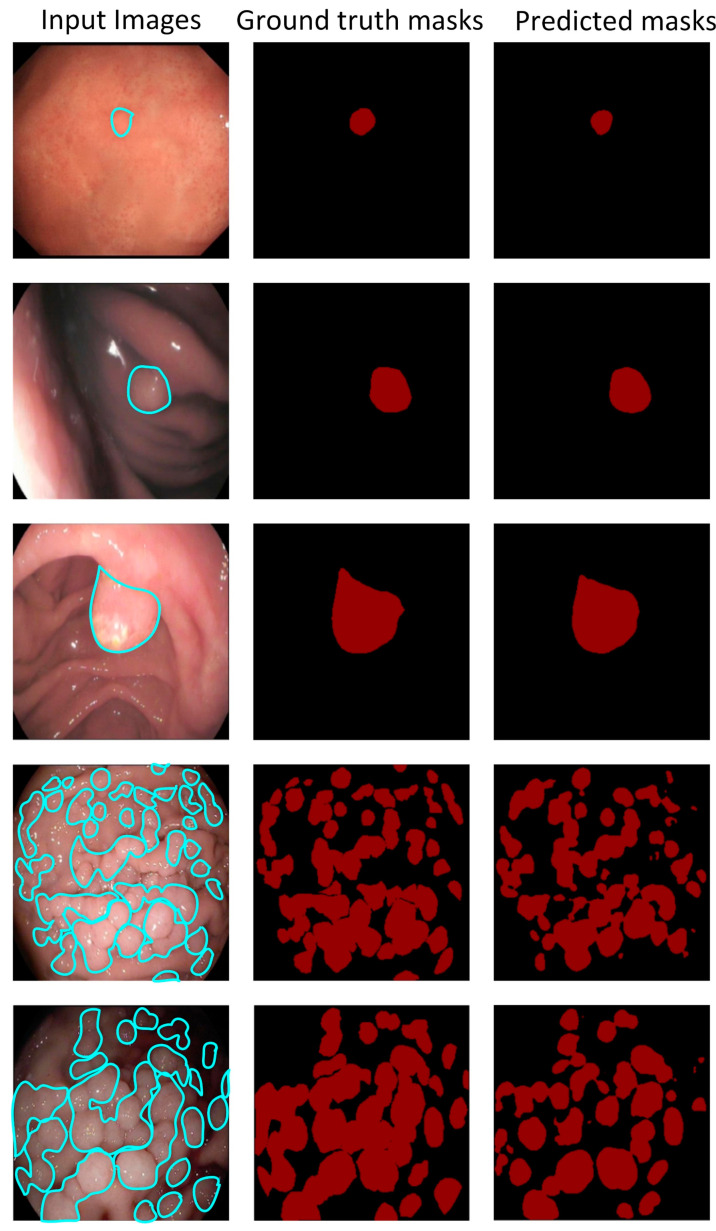
Sample results of the proposed automated poly-segmentation system.

**Figure 6:**
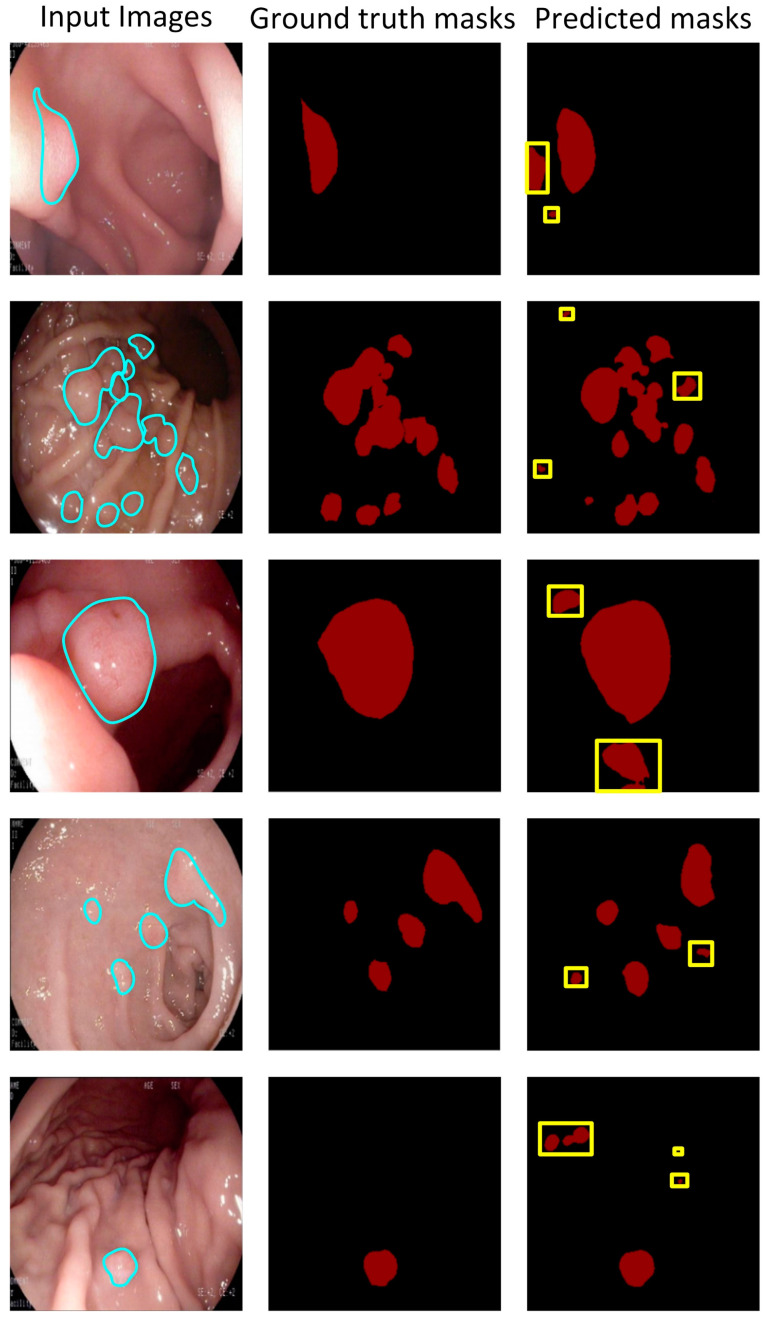
FP examples of the proposed automated poly-segmentation system.

**Figure 7:**
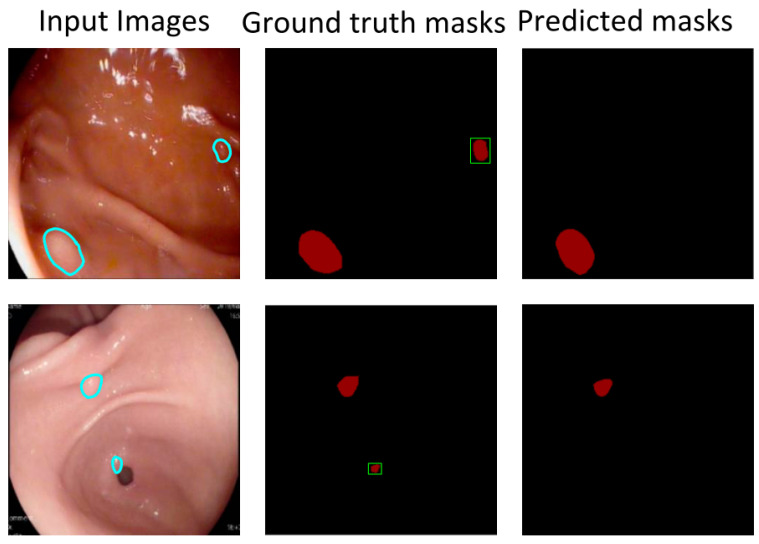
FN examples of the proposed automated poly-segmentation system.

Figure 5 shows some segmentation results from the “best score” model. The predicted masks have excellent locations and a fine-grained, well-segmented boundary regardless of small polyps, multiple polyps, or polyps in a dim background. It means that the pixels labeled as gastric polyps can be correctly classified. However, some pixels are misclassified. Figure 6 shows polyps are misclassified (i.e., the red area framed by the yellow box). As previously mentioned, the FP rate increases when the training data sets all contain gastric polyps. The reflective spots and gastric mucosal folds in the endoscopic images can easily be misdiagnosed as gastric polyps. Due to lighting or angles, these regions can indeed be easily misdiagnosed, even with the naked eye. Figure 7 shows that some polyps are missed by the system (i.e., the red area framed by the green box). These missed polyps are small polyps located at the edges of the images. Owing to hardware limitations, all endoscopic images are resized to 256 × 256, which affects the segmentation accuracy of small polyps. Even though there is room for improvement, the accuracy of the selected model is still very satisfactory.

All in all, the automated polyp-segmentation system may be beneficial clinically in three aspects, given its overall excellent performance. The system can play the role of a second observer by segmenting the detected polyps in real time on the monitor adjacent to the main monitor. It could be a useful tool for reducing skill differences among endoscopists and enhancing the quality of routine EGD. However, the final decision still relies on the endoscopist. Nevertheless, it can prompt the endoscopists for further processing, reducing the possibility of missing some polyps with the naked eye. For patients, the proposed system can improve the efficiency of physical examinations and shorten the time required for waiting and examination. Moreover, it can provide expert-level diagnosis, improving patients’ trust, cooperation, and satisfaction. Soon, this system will be open-source and free as compared to commercial software, making it very friendly for medical trainees and junior doctors to train and improve their clinical skills.

However, this system has a variety of limitations. First, it is only capable of segmenting gastric polyps and is not able to identify other gastric diseases simultaneously under EGD. Second, lesions are categorized as benign or malignant most of the time, which is difficult to distinguish under conventional white-light imaging endoscopy, and histopathological prognosis after identification can be more clinically valuable when combined with other advanced endoscopes. Thirdly, this system has not been subjected to multicenter clinical trials with large data sets, and it is still dubious whether it is robust enough to handle a large range of clinical uses.

## 5. Conclusions

Given the clinical impact of an increased gastric polyp detection rate on the incidence of gastric cancer, an automated polyp-segmentation system based on CNN is developed in this study to assist endoscopists in the diagnosis of gastric polyps, and a novel integrated evaluation approach is also proposed to select a CNN model with the best overall performance. In addition, some state-of-the-art semantic segmentation models are applied and compared, including U-Net, UNet++, DeepLabv3, DeepLabv3+, PAN, LinkNet, and MA-Net. Additionally, several advanced deep network architectures (i.e., ResNet50, MobileNetV2, and EfficientNet-B1) are used as encoders in these models. To evaluate the overall performance of semantic segmentation models, an integrated evaluation approach that combines the objective and subjective methods of MCDM is proposed. Eight metrics (i.e., IoU, accuracy, recall, precision, F1-score, model parameters, MACs, and FPS) are expanded from two dimensions (i.e., segmentation accuracy and computational efficiency). The metrics are condensed into the final scores to rank the candidate models by the CRITIC method and experts’ weight. The evaluation results show that UNet++ with the encoder of MobineNet v2 achieves the best scores in both subjective and objective as well as integrated evaluation approaches, although it is not the best on some single metrics. The proposed system achieves excellent performance in both segmentation accuracy and computational efficiency, contributing to the diagnosis and identification of gastric polyps. The automated polyp-segmentation system and the integrated evaluation approach are enlightening and show great potential in clinical applications. The originality of this study is summarized as follows:

(1) This study is pioneering research on gastric polyp segmentation. A high-quality gastric polyp dataset is generated. Seven semantic segmentation models are constructed and evaluated to determine the core model for the automated polyp-segmentation system.
(2) To comprehensively evaluate the results, the integrated evaluation approach combined with the CRITIC method and experts’ weight are combined to rank the candidate models, which is the first attempt at a polyp-segmentation task.

Although the above research results are remarkable, prospective research possibilities and potential enhancements are still needed to have better accuracy and efficiency in the detection of gastric lesions, which are listed as follows: (1) Clinically, the patient’s lesions are often more complex than a single lesion, such as chronic atrophic gastritis with intestinal metaplasia. For EGD, it will be more practical for the CAD systems to detect multiple lesions simultaneously. (2) Not only white-light imaging endoscopy but other more advanced endoscopes, such as narrow-band imaging and chromoendoscopy, should also be considered for the pathological prediction of gastric polyps. Histopathological prediction of small polypoid lesions based on identification allows the endoscopists to decide on a treatment plan once and for all to avoid progressive enlargement of polypoid lesions [52]. (3) Although the complex models show better performance in many cases, the high consumption of memory space and computational resources is an important reason that makes it difficult to implement them on various hardware platforms effectively. Thus, model compression and acceleration are the research directions for the deployment of the system; (4) The proposed integrated evaluation approach should be applied to more semantic segmentation tasks to evaluate its effectiveness, but not only for a one-time-used tool; and (5) The system should then be integrated into endoscopy equipment and used in clinical practice as the next step. The performance of the proposed system should be examined using multi-center and large-scale clinical data.

## Figures and Tables

**Table 1:** Constraints of the extant literature on gastric polyp segmentation employing CNN.

Reference	Dataset	Objective	Baseline	Constraint
Zhang et al. [18]	404 images	Real-time detection of gastric polyps	SSD	1. Most of the research focused on object detection, which is unable to portray the boundaries of polyps precisely. 2. The evaluation metrics are solitary without adequate consideration of clinical requirements.
Laddha et al. [19]	654 images	Detection of gastric polyps	YOLOv3 and YOLOv3-tiny	
Wang et al. [20]	1941 images	Detection of gastric polyps	Faster R-CNN	
Cao et al. [21]	2270 images	Stomach classification and detection of gastric polyps	YOLOv3	
Durak et al. [22]	2195 image	Detection of gastric polyps	YOLOv4, CenterNet, EfficientNet, Cross Stage ResNext50-SPP, YOLOv3, YOLOv3-SPP, Single Shot Detection, and Faster Regional CNN	

**Table 2:** Strengths and weaknesses of each model.

Model	Strength	Weakness
U-Net	Simple structure Less training data needed	Unknown depth of optimal architecture. Unnecessarily restrictive fusion scheme at the same-scale feature maps.
UNet++	A highly flexible feature fusion scheme Enhancement of segmentation quality of varying-size objects	Long time-consuming Easily prone to errors
DeepLabv3	A more general framework It can segment objects effectively at muti-scale.	Gridding Effect Problem Long-ranged information might be not relevant.
DeepLabv3+	Multiple effective field-of-view Results of shaper object boundary	Gridding Effect Problem Bilinear upsample cannot restore lost semantic information
PAN	It exploits global contextual information well Light and small computation	It needs numerous training data Difficult in locating small objects precisely.
LinkNet	Real-time oriented Simple structure and small computation	It needs numerous training data Lack of accuracy
MA-Net	It can capture rich contextual dependencies. Good performance	Heavy and expensive in computational resources Time-consuming

**Table 3:** Equations for metrics of segmentation accuracy.

Metrics	Equations
IoU	$IoU=\frac{\left|A\cap B\right|}{\left|A\cup B\right|}=\frac{\left|A\cap B\right|}{\left|A\right|+\left|B\right|-|A\cap B|}=\frac{TP}{TP+FP+FN}$
ACC	$\frac{TP+TN}{TP+TN+FP+FN}$
RE	$\frac{TP}{TP+FN}$
PR	$\frac{TP}{TP+FP}$
F1	$\frac{2\times PR\times RE}{PR+RE}$

Remark: ACC stands for accuracy, RE for recall, PR for precision, and F1 stands for F1-score.

**Table 4:** Weights of metrics.

First-Level Metrics	Second-Level Metrics	Weights
Segmentation accuracy	IoU	0.3
	Accuracy	0.05
	Recall	0.05
	Precision	0.05
	F1-Score	0.05
Computational efficiency	Number of parameters	0.1
	Number of MACs	0.1
	FPS	0.3
Sum of weight		1

**Table 5:** Workstation hardware and environmental configurations.

Configuration	Version
CPU	11th Gen Intel(R) Core (TM) i9-11900 @ 2.50 GHz
GPU	NVIDIA GeForce RTX 3080
RAM	64.0 GB
Operating System	Windows 10
Programing Language	Python 3.9
Frame	Pytorch-1.10.0
CUDA	11.4.1
cuDNN	11.4

**Table 6:** Test results based on the test dataset.

Model	Encoder	IoU (%)	ACC (%)	RE (%)	PR (%)	F1 (%)	No. of Parameters (M)	GMACs	FPS	CRITIC Score	Subjective Score	Final Score
U-Net	ResNet50	94.96	97.29	97.31	97.27	97.29	32.52	10.7	24	0.59	0.63	0.61
	MobileNet v2	95.56	97.56	97.57	97.55	97.56	6.63	3.39	26	0.75	0.81	0.78
	EfficientNet-B1	96.53	98.14	98.16	98.12	98.14	8.76	2.53	22	0.77	0.74	0.75
UNet++	ResNet50	96.57	98.18	98.20	98.16	98.18	48.99	57.54	20	0.53	0.53	0.53
	MobileNet v2	96.27	98.00	98.03	97.98	98.00	6.82	4.5	26	**0.84**	**0.88**	**0.86**
	EfficientNet-B1	96.79	98.11	98.14	98.09	98.11	9.08	5.1	21	0.73	0.70	0.72
DeepLabv3	ResNet50	96.23	97.95	97.98	97.93	97.96	39.63	40.99	24	0.65	0.70	0.67
	MobileNet v2	95.79	97.69	97.73	97.66	97.69	12.65	12.74	26	0.75	0.81	0.78
	EfficientNet-B1	95.46	97.37	97.40	97.34	97.37	9.81	3.37	23	0.63	0.65	0.64
DeepLabv3+	ResNet50	96.25	97.97	98.02	97.93	97.97	26.68	9.2	26	0.81	0.86	0.83
	MobileNet v2	95.22	97.36	97.42	97.32	97.37	4.38	1.52	**27**	0.75	0.82	0.78
	EfficientNet-B1	95.93	97.75	97.83	97.70	97.76	7.41	0.56	23	0.72	0.72	0.72
PAN	ResNet50	96.06	97.87	98.00	97.78	97.89	8.71	24.26	26	0.77	0.82	0.80
	MobileNet v2	95.17	97.27	97.50	97.14	97.31	**2.42**	0.79	26	0.72	0.77	0.75
	EfficientNet-B1	91.83	95.09	98.86	92.71	95.52	6.6	**0.09**	22	0.52	0.33	0.43
LinkNet	ResNet50	96.31	98.03	98.03	98.03	98.03	31.18	10.77	26	0.81	0.86	0.83
	MobileNet v2	95.11	97.32	97.33	97.32	97.32	4.32	0.94	26	0.71	0.77	0.74
	EfficientNet-B1	96.32	98.04	98.08	98.00	98.04	3.67	0.19	22	0.76	0.72	0.74
MA-Net	ResNet50	96.34	98.03	98.06	98.01	98.03	147.44	18.64	21	0.54	0.55	0.55
	MobileNet v2	96.23	97.98	98.00	97.96	97.98	48.89	5.27	24	0.74	0.76	0.75
	EfficientNet-B1	**96.57**	**98.15**	**98.16**	**98.14**	**98.15**	11.6	2.41	21	0.74	0.70	0.72

Remark: Boldface number means the best for each metric or score. ACC indicates accuracy, RE indicates recall, PR indicates precision, F1 indicates F1-score; Params indicates the number of model parameters in M. (1 M = 1 × 10^6^); GMACs indicates 1 × 10^9^ MACs; Subjective score indicates that the score is calculated by the subjective evaluation method.

## Data Availability

The datasets are available on reasonable requests from corresponding author.
